# “Lost trust in the system”: system barriers to publicly available mental health and substance use services for transgender women in San Francisco

**DOI:** 10.1186/s12913-022-08315-5

**Published:** 2022-07-19

**Authors:** Glenda N. Baguso, Karen Aguilar, Sofia Sicro, Malaya Mañacop, Jerry Quintana, Erin C. Wilson

**Affiliations:** 1 San Francisco Department of Public Health, San Francisco, USA; 2 University of California San Francisco, UCSF, San Francisco, USA; 3 Independent Consultant, San Francisco, USA

**Keywords:** Trans women, Qualitative, Mental health, And substance use services

## Abstract

**Background:**

Little is known about the barriers to mental health and substance use services for trans women living with HIV. We conducted a qualitative study with trans women living with HIV and providers to explore barriers to mental health and substance use services in San Francisco.

**Methods:**

We conducted focus group discussions and key informant interviews with a total of 15 medical, mental health, substance use, and social service providers and trans women living with HIV. We identified, analyzed, and reported themes using thematic analysis and derived themes directly from the data.

**Results:**

Our study participants identified two main themes and three subthemes. One main theme is that trans women and providers have lost trust in the system due to (a) lack of a linkage system between referrals and services, (b) structural barriers such as service location, language capacity, clinic hours, and (c) constant changes in services available. Another main theme is anti-trans and mental health stigma.

**Conclusions:**

Interventions to coordinate linkage from medical to mental health and substance use (MHSU) services are urgently needed to facilitate the utilization of MHSU services. Other interventions to improve quality monitoring and system improvement, and to address multiple stigmas broadly in society are needed to improve unmet MHSU service needs among trans women living with HIV in San Francisco.

## Background

Transgender (trans) women in San Francisco carry a large population-specific burden of HIV, with almost half estimated to be living with HIV as of 2020 [1]. Trans women in San Francisco have lower engagement in HIV care than other groups despite the availability of gender-affirming trans health clinics, HIV care experts, and universal health care access [2–4]. Trends in San Francisco are mirrored throughout the US. In a study of trans women receiving HIV care services throughout the country, fewer trans women had durable viral suppression than other populations living with HIV [3]. Viral suppression among trans women is 10% lower than in people living with HIV in San Francisco overall. COVID-19 has likely affected trans women’s viral suppression and HIV care engagement. As of March 2021, viral load tests, which are a marker of HIV care engagement, were down by 6% in San Francisco [2].

HIV care engagement and viral suppression are lower among trans women due to a myriad of factors rooted in discrimination toward trans women that leads to disproportionately high housing instability, food insecurity, criminalization, and violence [2, 4, 5]. Stressors from these experiences are associated with elevated mental distress and substance use, which impact HIV care engagement and outcomes [6, 7]. Many trans women living with HIV have trauma due to healthcare discrimination and may have high levels of anxiety, mistrust of providers, and fear of re-traumatization, which present barriers to initiating mental health and substance use (MHSU) services [8–11]. One of the many reasons for high rates of substance use among trans women is to cope with the effects of physical assault and intimate partner violence, which is a known barrier to adherence for those living with HIV [12, 13].

There is a considerable unmet need for substance use and mental health services for trans women living with HIV who access care in the public system [14]. And yet there is little data that exists on the barriers to substance use services and mental health support for trans women living with HIV. Our team conducted a qualitative study of the barriers to MHSU services, among trans women living with HIV. We aimed to engage providers and trans women to assess determinants that could be addressed in an intervention to improve mental health and substance use linkage for trans women living with HIV.

## Methods

The study included a convenience sample of providers and trans women living with HIV. We recruited providers for trans women living with HIV from eight organizations in San Francisco. Health care providers included a cisgender physician and cisgender nurses who specialized in HIV and gender-affirming care. Cisgender is a term used to describe people whose gender identity aligns with their assigned sex at birth. Mental health care providers consisted of licensed clinical social workers. Social service providers were trans and non-trans identified substance use program managers, case managers, and outreach workers. We also recruited trans women living with HIV who were not providers (n = 3). We hosted one focus group of staff from a gender-affirming HIV care clinic and conducted in-depth interviews with trans and non-trans-identified providers and trans women living with HIV.

Focus groups and in-depth interviews were conducted from February 2021 through May 2021 using semi-structured interview guides. Examples of focus group questions centered on exploration (i.e., Please describe the trans women patient population needs you primarily see at this clinic; What are the challenges with getting patients to initiate and stay engaged in mental health and substance use services at your clinic or when you refer outside your clinic?). For trans women living with HIV, examples of the interview questions centered on access (i.e., What are the main challenges for you when it comes to getting your health care needs met; How easy or hard was it to get access to mental health and/or substance use services?). Interviews were conducted over Zoom due to COVID safety protocols. All interviews were recorded and transcribed verbatim. Participants provided verbal informed consent. All methods were carried out in accordance with relevant guidelines and regulations. The study was approved by the Institutional Review Board at University of California, San Francisco (IRB#20-32166).

### Data analysis

To identify, analyze and report themes found in the data, we used an iterative approach in which thematic analysis was employed [15]. Due to the scarcity of studies regarding MHSU services for trans women, we used an inductive approach in which we derived categories and themes directly from the data. Coding was conducted in Dedoose (9.0.16) with one co-author (SS) and senior author (EW). Once coding was completed, we held weekly meetings which were attended by all authors. We began the process with data reduction, identified themes, deliberated on the conclusions, chose illustrative quotes, and wrote and edited the manuscript. Any concerns or conflicts were discussed on an iterative basis, and consensus was reached among the authors.

## Results

A total of 15 participants were interviewed. Professional roles and job titles are listed in Table 1. We found two main themes and three subthemes (Table 2). Barriers and facilitators to accessing MHSU services for trans women were discussed.

**Table 1:** Participant characteristics & roles

Characteristics & Roles	Frequency	Identified in paper
Cisgender HIV and gender affirming care specialist (*n* = 1) and cisgender nurses (*n* = 2)	*n* = 3	HIV care specialist
Transgender identified and cisgender program managers, case managers and outreach workers	*n* = 5	Social service providers
Cisgender licensed clinical social workers	*n* = 4	Mental health providers
Non-provider trans women living with HIV	*n* = 3	Trans women

**Table 2:** Main Themes and subthemes

Main Themes	Subthemes
Lost trust in the system	Suboptimal engagement to MHSU services due to the referral system
	Suboptimal engagement to MHSU services due to structural barriers
	Suboptimal engagement to MHSU services due to constant changes within the existing system
Anti-trans and Mental Health stigma	

### Main theme: Lost trust in the system

A main theme identified was that patients and providers had lost trust in the system’s ability to best meet trans women’s needs for MHSU services. Providers explained that access to publicly available MHSU services was mostly facilitated through referrals from primary medical care providers. Although trans women could initiate engagement in services on their own, most trans women living with HIV were linked to MHSU services via their HIV specialist and gender-affirming primary care providers. MHSU service barriers were linked to barriers to medical care, and medical care was a key access point to obtaining publicly available MHSU services for trans women living with HIV in San Francisco.

#### Subtheme: suboptimal engagement to MHSU services due to the referral system

A recurring system barrier was the referral system for MHSU services. Participants explained that MHSU referrals were primarily facilitated by medical providers in their gender-affirming primary medical care setting. Trans women reported that it took multiple medical clinic visits to receive an MHSU referral. When referrals were made, the onus of follow-up to initiate a new service was the responsibility of trans women living with HIV. Lack of follow-up put the responsibility of initiating MHSU services on the patient, which lowered the chance of follow-through to initiation.

Providers reported that service initiation was a laborious process, as there were multiple steps in the intake process before people would see a counselor.

I do think there actually is a wide disparity in terms of how intake is done, and this is another problem, actually, because primary care clinics need to be aware of how each clinic might operate a little bit differently, which is kind of a crazy thing to ask them to know (mental health provider A).

Additionally, providers at the medical clinic were not aware of what intake processes were in place, so they did not prepare nor support patients through the intake process.

#### Subtheme: suboptimal engagement to MHSU services due to structural barriers

Providers and trans women living with HIV described structural barriers to medical, substance, and mental health care. Safety, clinic reputation, language, clinic hours, and lack of trans-specific clinics and trainings may have impacted visit engagement and access to MHSU services. Providers discussed that some medical and social services had relocated to unsafe neighborhoods in San Francisco. Clinic location was especially important for those who were in recovery or struggling with substance use. Social service provider D stated,

It’s right there in a drug-infested area…… People are harassed because when I used to go there, I’d come out of the doctor’s office, ‘You got these kind of pills or those kind of pills?’ It’s like, ‘Sweetie, come on. Leave me alone.’

Visit attendance among those in recovery and struggling with reducing substance use may have been impacted by having to access medical care in locations where active drug selling was regularly occurring. Providers also speculated that a clinic’s reputation may determine if and where trans women would seek treatment. Providers reported that when it is known that a clinic provided poor health care to the trans community, it impacted engagement with the MHSU services.

Structural barriers related to language capacity and hours in which services were offered were also important barriers. Some providers discussed that there were not enough Spanish speakers in MHSU services, which created access barriers for mono-lingual Latina trans community members. Other providers discussed that there were no after-hours MHSU services known to them. Furthermore, some clinics and programs that offered MHSU services, struggled with providing trans-competent care, which participants said was exacerbated by COVID-19 as many providers left the field and services were shuttered. Thus, trans women living with HIV were only offered services with the general public. Social service trans-identified provider B stated,

“I mean, they don’t want to go to the [location] because they don’t feel comfortable. …. And I mean, it’s like, for me, I always felt that uncomfortable feeling because I was like, most of the meetings, I was the only trans person there.”

Providers also said they did not receive medical, mental health, or substance use training on how to provide gender-affirming services and learned about the needs of trans women living with HIV on the job. Providers mentioned that even when they were trained on the job, many other people in the clinic, from security to benefits specialists, did not have training on gender-affirming care. This was a concern as providers reported that there is no routine oversight or monitoring of the quality of services trans people in the system receive.

#### Subtheme: suboptimal engagement to MHSU services due to constant changes within the existing system

Another important subtheme was that constant changes in what was offered in the public system created distrust and barriers to engaging in services. One participant reported that a side effect of innovations in services and changing city priorities was a lack of consistency that left some key populations, including trans women, behind. Social service provider A stated,

The truth is, in San Francisco, a lot of people have lost trust in the system because we’ve had so many different programs and we’ve been so innovative in trying to find the right fit for the community that a lot of people got stepped on and left out in the process.

Constant changes in treatment programs had a negative impact on community members’ interest in MHSU services. Changes at the clinic level created instability that reduced patient satisfaction. It is worth noting that some substance use services struggled to meet the needs of trans women living with HIV. Providers discussed that other barriers were shortened treatment times in substance use programs from 6-months of detox and inpatient rehabilitation to 30, 60, and 90 days. Decreasing the days of treatment was seen as less efficacious for substance use recovery abstinence outcomes and may impact post-treatment care of their patients. People who completed their treatment in 3 months often returned to the same neighborhood.

…then, the panic comes in when the treatment is over of where am I going? I’m going right back to the same neighborhood where I was at. It’s like nothing’s going to change. … Something needs to happen where you start making a way for people to not live in that area, if possible … Maybe they need more housing programs (social service trans-identified provider A).

Providers reported that trans women recovering from substance use needed long-term stable housing to increase their chances of recovery.

### Main theme: anti-trans and mental health stigma

Participants reported that they observed and experienced anti-trans discrimination in the San Francisco public healthcare system. Social service trans-identified provider C recalled a waiting room in a clinic where the provider overheard a staff member deadnaming a trans client.

She (staff) was calling the patient by their male name. And then when I was talking to her, the patient, was like, ‘I don’t care.’ But I know they care. I know in the back of their head they care. They just don’t have the energy to fight every single day.

Because of the tether between medical care and other services via the referral system, healthcare avoidance from discrimination had a direct impact on MHSU access. The vicarious trauma providers experienced from serving the trans community may also lessen trans provider’s interest in pushing for referrals.

An important barrier to engaging in mental health services specifically was the stigma associated with engaging in therapy. One trans woman living with HIV said, “I think people have this notion that if you say, ‘Oh, I see a therapist’ that means you’re cray cray.” Mental health provider A said,

Related to that is I think there’s the issue of stigma at a mental health clinic and seeking out mental health services as a trans person that is just really…I think it’s a really big barrier...

The issue of mental health stigma hinders engagement to MHSU service among a community already burdened with multiple stigmas due to their gender identity and/or race.

### Facilitators and recommendations

Providers recommended that a peer navigator could be hired to facilitate access to MHSU services through the fractured public health system and help rebuild trust in the system. Furthermore, a support group may reduce the heightened social isolation trans women experience due to COVID-19. Providers suggested that peer support groups would be helpful for people in recovery from addiction. “I think it would be good to have a group with maybe, I would think maybe once or twice a month to have guest speakers that share their experience of how life was for them (social service provider B).”

Trans health care needs, such as any gender-affirming procedures were seen as a facilitator in seeking mental health services. In San Francisco, residents’ gender-affirming procedures are covered if they are eligible for Medi-Cal or Healthy San Francisco, the city’s health access program.

I think that there’s a way in which I think the surgery thing and the mental substance use actually runs very much in parallel, and that could be an incentive for someone to agree to uptake a navigator because this is a person who’s going to help you meet the requirements that you need to get your electrolysis to get your surgery (HIV care specialist).

While other providers agreed, there was discussion of the impact on the client’s comfort level and the necessity of overcoming that discomfort in order to engage in gender-affirming surgeries.

Finally, our providers highlighted the importance of making connections between life stressors, mental health, and substance use as knowledge that could promote interest and engagement in MHSU services. One HIV care specialist stated,

...I think that one of the things I see as a need is that for the particularly trans women clients that we have, the amount of substance use is really high. And not surprisingly, we see people who are socioeconomically disadvantaged, traumatized, etc. So, a lot more people are going to use to cope.

Lack of knowledge about the root causes of their mental distress or substance use may influence their engagement or lack of engagement to MHSU services. Increasing this knowledge through peer navigation, support groups and self-advocacy may potentially increase MHSU engagement.

## Discussion

We found substantial structural and systems barriers that may contribute to unmet MHSU needs for trans women living with HIV in our city. Our medical services are intricately connected to MHSU services through the referral system, thus barriers that impact the medical services, in turn, impact access to MHSU services. One of the most common findings was that linkages between referrals and services were broken. Participants reported that referrals for MHSU services were often made without follow-up to ensure services were initiated. Providers in the medical setting neither had the time nor the resources to ensure referrals converted to services initiation. This is consistent with another study finding that lack of follow-up on referrals, in addition to complex referral systems and compartmentalization of specialty services, were barriers to health care utilization among trans women living with HIV in Canada [16].

Another system barrier was the availability of ancillary services that were needed to support recovery from substance use and promote mental health. For example, even after trans women were linked to substance use treatment services and completed a program, many faced unstable housing that often resulted in relapse as a way to cope with unmet basic needs. Stable housing has been identified as a crucial pillar to recovery [17]. The need for services to provide access to housing, opportunities for jobs, and safety, are likely needed before trans women living with HIV can be expected to initiate and use MHSU services.

Barriers to services also existed because of the constant changes in the system of services. Participants described how innovations in services, relocation of clinics, staff changes, and other clinic operation changes resulted in confusion about what services were currently available. Consistent with past literature, safety concerns related to their medical clinic’s location and the clinic’s patient population were a deterrent to accessing care [18]. The lack of knowledge and misinformation underscores the need for better communication between different clinics and providers in the public health system. Past studies indicate that organizational changes such as staff turnover and management reorganization were associated with poor patient outcomes and a decrease in the effectiveness of treatment among patients with personality disorders [19]. However, when health providers are involved in these changes, and these changes are communicated, successful organizational change can occur and continuity of care for patients can be achieved [20, 21].

We also identified barriers that may impact trans women’s willingness, motivation, and readiness to participate in MHSU services. Providers discussed how poor mental health hindered their client’s ability to engage in their MHSU services. Trans women living with HIV may lack knowledge about the root causes of their mental distress or substance use and the services offered to address these issues. Other studies support that people may lack insight into how daily stressors can impact their mental health and their need to engage in mental health services [22, 23].

Anti-trans stigma within the system and fear of mental health stigma were also important barriers to MHSU service. Fear of discrimination due to gender identity and HIV stigma have led to delays in health and health care among trans women [9]. Mental health stigma, another stigma that trans women living with HIV in our study expressed, is a main barrier to seeking mental health services among trans people [24].

Several limitations of this study should be noted. This study was conducted with trans women, trans-identified providers, and cisgender providers in the public health systems. However, there were only a small number of trans women interviewed. This study relies primarily on the views of trans-identified and cisgender providers. Providers from a private health system or those in acute care settings may provide different perspectives and experiences in the referral process for trans women living with HIV. Furthermore, as San Francisco offers universal health care, these findings may not apply to other geographical areas. We also note that due to isolation, uncertainty, and in the context of the pandemic, an increase in poor mental health puts a strain on an already loaded mental health system [25]. Barriers may likely have increased, and new barriers may result from a shortage of staff and other resources.

### Recommendations

Interventions are needed to mitigate the systems, stigma, and structural barriers trans women living with HIV face in initiating and engaging in MHSU services. Perhaps the most important intervention needed is the development of a coordinated MHSU linkage system that helps address concerns and facilitates access for trans women living with HIV to engage in mental health and substance use services. Participants reported that peer navigation may help facilitate linkages and help address barriers. Navigation services have been successful in engaging trans women in HIV care [26]. Navigation services, and particularly peer navigators who identify as trans and work in the public health system have been shown to improve HIV care engagement and outcomes and may provide the needed bridge for trans women to link to MHSU services through the fostering of trust, the similarities in background between peer and participant and accessibility of peer navigators [26–29]. Not only can peer navigators offer support through referrals to ancillary support programs, but they can educate, provide feedback, and motivational interventions to teach about the connection between social and daily stressors with mental health and substance use and may create motivation to engage in MHSU services [28, 29].

Peer navigators need institutional support in which gender-affirming trainings for staff are tracked and supervised and a professional support system that has a process to discuss opportunities to decrease stigma and discrimination in the workplace and with the trans community. Monitoring to ensure quality services are being offered may be supportive of the work of navigators. Having peer navigators and more staff and health care providers who are from the trans community may help address quality concerns and mitigate anti-trans stigma still existing in the public health system while empowering trans women living with HIV and providing the impetus to engage in MHSU services. Increasing the number of trans-identified health care providers needs a substantial increase in education and employment opportunities for the trans community. We recommend that additional funds and resources be allocated to increase the number of trans people in the public health workforce.

## Conclusions

Health care providers identified several barriers to mental health and substance use service for trans women living with HIV in San Francisco: the referral system, location of clinic, clinic hours, clinic reputation, lack of trans-specific clinics and programs, changes within the public health system and anti-trans and mental health stigma. These findings suggest that there is a need on multiple fronts to help trans women utilize mental health and substance use services. A peer navigator may help trans women navigate the health care system and link them to mental health and substance use services.

## Data Availability

The datasets used and/or analysed during the current study available from the corresponding author on reasonable request.
